# Tracheal Tissue Engineering: Advances and Challenges

**DOI:** 10.3390/bioengineering13060641

**Published:** 2026-05-29

**Authors:** Nina D. Kosciuszek, Joanne Walker, Heather Wanczyk, Christine Finck

**Affiliations:** 1 Division of Pediatric Surgery, Connecticut Children’s Medical Center, Hartford, CT 06106, USA; nkosciuszek@uchc.edu (N.D.K.); jowalker@uchc.edu (J.W.); wanczyk@uchc.edu (H.W.); 2 Department of Pediatric Surgery, University of Connecticut School of Medicine, Farmington, CT 06030, USA

**Keywords:** tracheal tissue engineering, tracheal regeneration, biomaterials, regenerative medicine, scaffold biomechanics

## Abstract

Traumatic tracheal injuries and congenital defects can be life-threatening and are associated with substantial morbidity and mortality. Regenerating the trachea through tissue-engineered scaffolds has emerged as an innovative alternative to traditional therapies that involve tracheal resection with primary end-to-end anastomosis or tracheostomies. Despite significant advances in biomaterial developments, stem cell biology, and novel scaffold fabrication, successful clinical translation of tracheal constructs remains limited. Major challenges include inadequate vascularization following implantation, epithelial regeneration, immune reactions, mechanical instability, infection, and inability of adaptive scaffold systems to withstand long-term tissue remodeling. While general tracheal tissue-engineering techniques and the materials, cell lines, and fabrication methodologies have been previously explored, this review summarizes current advancements in tracheal tissue engineering while emphasizing the mechanobiological and translational barriers that preclude functional tracheal regeneration and clinical success. Emerging knowledge in immunomodulatory biomaterials, dynamic scaffolds, strategic vascularization methods, and adaptable constructs has paved the way for researchers to develop a tracheal scaffold that can be translated into clinical use. This review provides a critical framework that discusses the advantages and potential pitfalls of the aforementioned technologies.

## 1. Introduction

Traumatic tracheal injuries and congenital airway defects in adult and pediatric populations remain difficult to manage with high morbidities and mortalities [1]. Most tracheal injuries are iatrogenic, although they may also result from blunt or penetrating trauma [2]. Additional tracheal abnormalities arise from congenital defects such as severe laryngotracheal clefts (type IV), which are rare but fatal without intervention [3]. Extensive tracheal defects following oncological resection also remain challenging, as current surgical techniques cannot reliably reconstruct surgical defects exceeding 40–50% of the tracheal length in adults and 30% in children [4]. Together, these limitations highlight the urgent need for regenerative strategies capable of restoring functional tracheal tissue through tissue-engineered scaffolds.

When surgical repair is feasible, approximately 20% of patients experience complications, such as granulation tissue formation, tracheal stenosis, anastomotic separation, tracheoesophageal fistula (TEF), tracheoinnominate fistula, and impaired mucociliary clearance [5,6,7,8,9]. Nearly 4% of patients who develop symptomatic anastomotic stenosis require multiple additional procedures such as balloon dilation or even reoperation [6,7]. These complications significantly impair quality of life and increase healthcare utilization, emphasizing the need for improved regenerative approaches that promote physiologic tissue healing and remodeling.

Despite decades of investigation, successful clinical translation of tracheal tissue-engineered scaffolds remains limited. Early clinical attempts highlight significant challenges, including scaffold ischemia, infection, inadequate epithelialization, and fibrosis, which may contribute to scaffold failure [8]. Highly publicized failed attempts at tracheal regeneration illustrate consequences of poor vascular supply, insufficient preclinical studies, and incomplete understanding of long-term changes from host immune responses and tissue remodeling [10,11]. These experiences underscore the complex interplay that exists between scaffold biomechanics, vascularization, epithelization, and immune-mediated tissue remodeling that needs to be addressed in order to achieve functional airway regeneration with engineered constructs [12].

Several recent reviews have explored scaffold fabrication techniques, biomaterials, and cellularization strategies in tracheal tissue engineering [6,13,14]. This review summarizes recent advances in tracheal tissue engineering while focusing on the mechanobiological and translational barriers limiting functional airway regeneration.

## 2. Native Tracheal Biology and Implications for Tissue Engineering

Understanding the embryology and anatomy of the trachea is essential for the development of functional engineered tracheal constructs. The trachea serves as a crucial conduit for airflow during respiration while simultaneously regulating humidity, temperature, and defense against inhaled pathogens and debris [15]. During embryogenesis, the trachea and esophagus arise from a common foregut tube through coordinated epithelial–mesenchymal interactions regulated by multiple signaling pathways [10,11]. These developmental processes continue to influence tissue repair following injury later in life [10,16].

**Figure 1:**
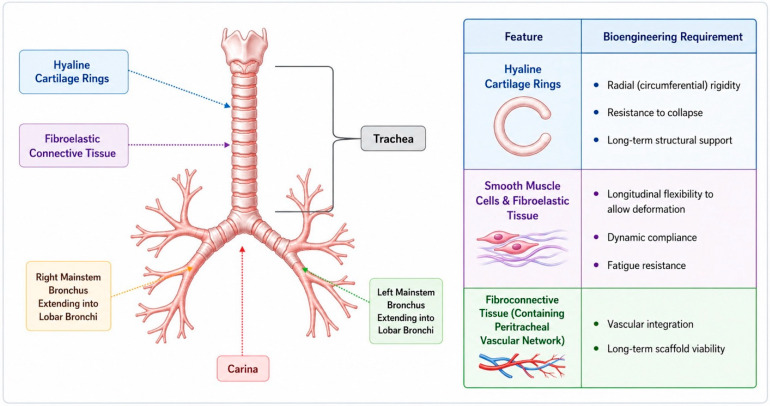
Tracheal anatomy and associated biomechanical design requirements for tracheal tissue engineering. Hyaline cartilage rings provide rigidity while maintaining patency, while the posterior smooth muscle and fibrous connective tissue preserve flexibility during respiration and cervical spine motion. Original image created in BioRender and then visually enhanced with the assistance of ChatGPT. Created in BioRender. Kosciuszek, N. (2026) https://BioRender.com/s2xo1f8 (accessed on 16 May 2026).

The trachea is composed of 16–20 hyaline cartilage rings located on the anterior and lateral walls with a layer of smooth muscle posteriorly, producing the characteristic U-shape [15]. Fibroelastic connective tissue bridges adjacent cartilage rings and is continuous with the perichondrium [17]. Surrounding the trachea is a loose fibroconnective areolar tissue that contains a network of blood vessels and nerves [17]. This highly specialized architecture provides circumferential rigidity while preserving flexibility during respiration, coughing, swallowing, and cervical spine motion. Replicating these biomechanical properties remains one of the major challenges in tracheal tissue engineering. Figure 1 demonstrates basic tracheal anatomy with a breakdown of key anatomic features and the associated mechanobiological engineering requirement. Figure 1 demonstrates basic tracheal anatomy with a breakdown of key anatomic features and the associated bioengineering requirement.

**Figure 2:**
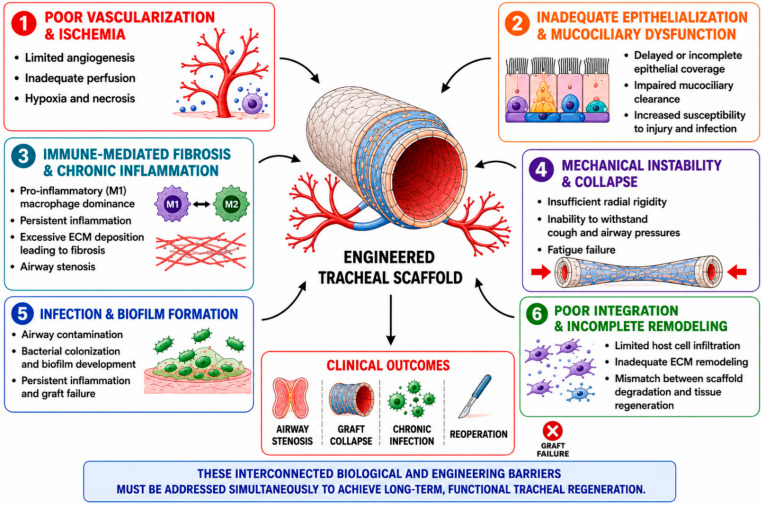
Major biological and engineering barriers limiting successful tracheal regeneration following implantation of tissue-engineered airway scaffolds. Key challenges include inadequate vascularization, delayed epithelialization, immune-mediated fibrosis, mechanical instability, infection and biofilm formation, and poor host integration. Together, these interconnected barriers contribute to airway narrowing, chronic infection, graft collapse, and long-term graft failure, highlighting the need for multifunctional scaffold systems capable of supporting vascularization, epithelial regeneration, immunomodulation, and biomechanical stability. Detailed ideas and instructions were entered into ChatGPT for assistance in creating this figure. The authors reviewed the figure and agreed with the information provided.

Successful tracheal regeneration requires coordinated restoration of epithelial function, vascularization, immune compatibility, mechanical stability and long-term tissue remodeling (Figure 2 and Table 1). Each of these barriers must be addressed to achieve reliable airway repair and regeneration. Unlike many other tissue-engineered systems, tracheal scaffolds function within a dynamic airway environment exposed to repetitive mechanical loading, airflow shear stress, mucous accumulation, and microbial colonization. Consequently, failure of a single regenerative component may compromise overall graft integration and long-term airway patency.

**Figure 3:**
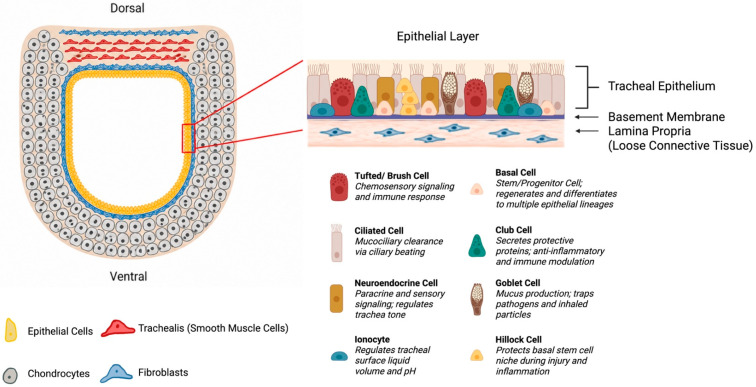
Cross-section depiction of trachea, highlighting the intricate layers of chondrocytes, fibroblasts, smooth muscle cells, and epithelial cells. The zoomed-in panel depicts cells that make up the epithelial layer. The original tracheal cross-section was drawn and then modified with ChatGPT to remove imperfections. That image was then uploaded to BioRender for further production. Created in BioRender. Kosciuszek, N. (2026) https://BioRender.com/7g7t7sd (accessed on 16 May 2026).

The tracheal epithelium serves as both a physical and immunological barrier and is essential for mucous production, mucociliary clearance and airway homeostasis [15,34,35,36]. The epithelial layer is composed of ciliated, basal, goblet, club, neuroendocrine, tuft, and hillock cells, each with specialized functional roles (Figure 3 and Table 2) [16]. Inadequate epithelialization following scaffold implantation may result in impaired mucous clearance, chronic inflammation, bacterial colonization, and airway obstruction.

Basal cells function as resident stem/progenitor cells capable of self-renewal and differentiation into multiple epithelial cell populations following injury [26,28,29,30,32,33,34,35]. Two types of cells that basal cells differentiate into are goblet and ciliated cells (Table 2) [26,28,29,30,32,33,34,35]. Consequently, current regenerative approaches increasingly focus on epithelial progenitor cell delivery, stem cell seeding, airway organoids, and bioactive scaffold coatings that promote epithelial attachment and differentiation [28,38]. However, achieving long-term functional epithelial maturation and coordinated mucociliary activity remains a major translational challenge.

Surrounding the epithelial layer is the basement membrane, followed by smooth muscle (posteriorly), fibrinous connective tissues containing fibroblasts, and cartilage composed of chondrocytes (anteriorly) (Figure 2) [16]. The basement membrane assists in structural integrity and filtration of different cell types through pores, such as various immunogenic cells [39]. Immune compatibility and regulation of the foreign body response (FBR) represent a major challenge to successful scaffold integration. Implanted biomaterials can trigger persistent inflammatory responses characterized by macrophage activation, giant cell formation, fibrosis, and excessive extracellular matrix deposition that contribute to stenosis and graft failure [18]. In addition, the airway is continuously exposed to inhaled pathogens and bacterial biofilms, which further complicates long-term scaffold survival. Emerging immunomodulatory strategies focus on promoting constructive tissue remodeling through biomaterial surface modification, cytokine delivery, extracellular vesicles, and scaffold topographies that favor pro-regenerative M2 macrophage polarization while reducing fibrotic encapsulation triggered by pro-inflammatory M1 macrophage polarization [6,18,36].

The trachea is subjected to cyclic compression and expansion during respiration, rapid changes in pressure, and multidirectional cervical movement while maintaining a delicate balance between rigidity and pliability [24]. Mechanical stability provided by the cartilaginous rings is important for maintaining airway patency and preventing collapse during respiration [24]. In situations where forceful coughing is required, smooth muscle cells assist by pulling the cartilage rings closer to each other, constricting the airway while preventing collapse [40]. Mechanical characterization studies have demonstrated age-dependent differences in tracheal stiffness, with increased rigidity observed in older patients (20.5 pascals) compared to younger patients (12.2 pascals) [15,24]. This knowledge is important for scaffold design as mechanical mismatch between engineered constructs and native tissue may contribute to scaffold collapse, narrowing, impaired epithelial integration, and chronic inflammation. In addition, insufficient radial rigidity may result in collapse, while excessive stiffness may impair tissue healing and alter airflow. To address these challenges, current engineering approaches utilize a variety of materials to add stability and better replicate native tracheal biomechanics [6,41,42,43,44]. However, achieving long-term fatigue resistance and mechanical durability while simultaneously supporting cellular integration and scaffold degradation remains an ongoing challenge.

When considering tracheal regeneration for diverse conditions ranging from congenital to acquired injuries, it is important to recognize the anatomical and functional differences between pediatric and adult patients [45]. In children, the trachea is shorter, narrower, and more compliant and has more tracheal rings compared to adults (newborns: 10 rings; adolescents: 8 rings; and adults: 6 or fewer from the sternal notch) [45,46,47]. For the pediatric population, scaffold development needs to account for increased growth over time, while maintaining structural integrity and functional properties, so that the engineered tissue will develop with the individual throughout adulthood [45,46,47]. Emerging approaches, including biodegradable scaffolds and mechanoresponsive biomaterials, aim to create constructs capable of accommodating continued tracheal growth while preserving integrity and tracheal patency [36].

Collectively, these biological and engineering barriers highlight the complexity of achieving functional tracheal tissue regeneration. Successful clinical translation will likely require integrated multifunctional scaffold systems capable of providing mechanical stability, cellular integration, and epithelialization while enabling flexibility and growth.

## 3. Cell Types Used in Tracheal Tissue Engineering and Regeneration

Cell-based therapies play a central role in tracheal tissue engineering as successful airway regeneration requires coordinated restoration of epithelial, cartilaginous, vascular, and immunologic function. Multiple cell populations have been investigated, including mesenchymal stem cells (MSCs), induced pluripotent stem cells (iPSCs), airway basal stem cells, endothelial cells, and primary chondrocytes. Each cell type offers distinct regenerative advantages while also presenting unique translational limitations that influence scaffold integration, tissue remodeling, and long-term graft functionality.

MSCs remain one of the most extensively studied cell types in tracheal tissue engineering and regeneration because they are readily accessible, easily harvested from various tissues (bone marrow, adipose, placenta, and umbilical cord), multipotent (cartilage, bone, and adipose tissue), immunomodulatory, and capable of promoting angiogenesis and tissue repair [48,49]. Given this, many researchers utilize MSCs in their scaffold design for tracheal regeneration [49]. Shin et al. cultured autologous MSCs on a porcine cartilage powder-based tracheal scaffold that was then implanted into tracheal defects in rabbits and saw development of intact respiratory epithelium and patent luminal contour at the site of transplantation [50]. It is theorized that MSCs’ long-term regenerative contribution may rely heavily on paracrine signaling and immune modulation rather than direct tissue replacement.

Induced pluripotent stem cells (iPSCs) derived from fetal or adult somatic cells have emerged as a promising platform for patient-specific tracheal regeneration. Somatic cells are reprogrammed by either a viral or mRNA-mediated process into iPSCs, which can be differentiated into different cell lineages using specific growth factors for the cell type of interest [51]. For example, iPSCs can be programmed to differentiate into tracheal epithelial and cartilaginous cells using lineage-specific differentiation protocols incorporating growth factors. These cells are beneficial as they have unlimited proliferative capacity and can be patient-derived for autologous administration, thereby reducing immunologic incompatibility and mitigating ethical concerns associated with embryonic stem cells [51]. In one study, iPSCs were differentiated into chondrocytes, seeded onto a 3D scaffold and implanted into a tracheal defect in nude rats. Cartilage-like tissue was observed in the regenerated tracheal wall at 4 weeks [19]. Another similar study identified functional ciliated epithelial cells on the luminal side of their scaffold seven days after implanting differentiated iPSCs into rat tracheas [21]. iPSCs have significant potential due to their pluripotency; however, their use remains limited due to concerns of tumorgenicity, genomic instability, inconsistent differentiation efficiency, and difficulty generating enough cells to accommodate complex large-scale manufacturing.

Terminally differentiated, primary cell populations have also been investigated to improve structural and functional tissue regeneration from scaffolds. Chondrocytes are commonly studied due to the support provided by cartilaginous tracheal rings. Nomoto et al. developed a polypropylene tracheal prosthesis seeded with chondrocytes and implanted into rabbit tracheas with resultant cartilaginous tissue formation after fourteen weeks [22]. More importantly, the tracheal shape and structural integrity were preserved [22]. Although primary chondrocytes provide strong cartilage-forming potential, their clinical use may be limited by donor site morbidity, restricted proliferative capacity, and phenotypic instability during in vitro expansion.

More recently, there has been a focus on primary airway-specific progenitor cells, including basal epithelial cells or endothelial cells. As previously described, tracheal basal stem cells are capable of self-renewal and differentiation into multiple respiratory epithelial subtypes of the pseudostratified epithelium [19,26,28,29,30,34,35,52], which makes them an attractive cell source for the restoration of functional mucociliary epithelium. However, maintaining their self-renewal capacity in vitro is difficult, which limits their widespread use. Endothelial cells are also important because vascularization remains one of the major limitations preventing long-term graft survival and integration [21]. The incorporation of endothelial cells into scaffold systems may improve angiogenesis and microvascular stabilization, which can then improve oxygen and nutrient delivery. Nevertheless, endothelialized and epithelialized scaffolds require highly supportive microenvironments and synchronized signaling networks to maintain the vascular bed.

Collectively, no single cell type currently satisfies all biological and translational requirements necessary for tracheal regeneration. Based on preliminary pre-clinical studies, MSCs provide immunomodulatory and anti-inflammatory benefits, whereas iPSCs offer greater differentiation potential and patient specificity but raise concerns regarding tumorigenicity and clinical safety. Tracheal progenitor cells and chondrocytes also contribute specialized regenerative functionality with a focus on targeted cell types. Future regenerative strategies will likely rely on multicellular scaffolds capable of simultaneously supporting epithelialization, vascularization, immune modulation, and tissue remodeling. Table 3 summarizes commonly studied cell types used in tracheal tissue engineering along with their advantages and limitations.

## 4. Biomaterials Used in Designing Scaffolds for Tracheal Tissue Engineering and Regeneration

Scaffolds are based on a three-dimensional template that provides a structural, mechanical, and biological framework necessary to support cell infiltration, growth, and proliferation. Scaffolds composed of various biomaterials have demonstrated promise in tracheal regeneration and tissue engineering. Biomaterials used for tracheal tissue regeneration are classified as natural, synthetic, or hybrid (Table 4).

Natural biomaterials, including alginate, chitosan, collagen, fibrin, gelatin, hyaluronic acid, and soy protein, possess favorable biological properties due to their biochemical similarity to native extracellular matrix composition [6,23,25,53,54,55,56]. These materials support cellular adhesion, migration, proliferation, and tissue remodeling while promoting a favorable regenerative microenvironment [6]. For example, collagen–hyaluronic acid scaffolds seeded with human umbilical vein endothelial cells and MSCs have been shown to promote vascular development in a chick chorioallantoic membrane model [6,8]. While this study did not implant these scaffolds in vivo, these results are still important as this study illustrates the ability of collagen and hyaluronic acid promoting vascularization and tissue remodeling. Furthermore, natural biomaterials may contain intrinsic bioactive motifs and proteins that regulate stem cell differentiation, epithelial maturation, and immune signaling. These results are particularly important because insufficient vascularization remains one of the primary limitations in tracheal regeneration [6,57]. Natural biomaterials have limited mechanical strength and rapid degradation kinetics, making them vulnerable to deformation and breakdown under the mechanical stresses experienced within the trachea [6]. One way to circumvent this limitation is through the use of stronger synthetic materials.

Synthetic biomaterials provide improved mechanical strength, tunable degradation kinetics and reproducible manufacturing properties [6]. Commonly investigated synthetic polymers are listed in Table 4. Biodegradable polymers such as PLA, PCL, PGA, and PLGA are attractive polymers to work with as degradation profiles can be modified, which allows researchers to control tissue regeneration and degradation timeline of the scaffold [6,42,43,52]. Additionally, advances in additive manufacturing with three-dimensional (3D) bioprinting of synthetic materials now allow for fabrication of patient-specific scaffold architectures with mechanical properties that more closely represent native tracheal mechanical properties.

Despite these significant advantages, a drawback to synthetic polymers is their limited biocompatibility, which can cause an unwanted immune response and possible rejection [6]. Scaffolds composed of synthetic biomaterials may alter local immune homeostasis as the immune cells may recognize the synthetic material as a foreign body; therefore, provoking an inflammatory response that can lead to scaffold rejection [18,58]. Also, synthetic polymers are generally bioactively inert and hydrophobic, which can make cell attachment difficult [6,52]. Moreover, degradation byproducts from synthetic polymers can promote inflammation or acidic microenvironments that can further impair scaffold acceptance and incorporation [6,52].

In an effort to optimize the strength of natural and synthetic materials, hybrid scaffolds combine the biological advantages of natural materials with the structural strength of synthetic polymers. Researchers are developing hybrid biomaterials to improve bioactivity, provide epithelial support, and promote biocompatibility with sufficient mechanical strength to withstand physiological forces. One study developed a hybrid scaffold composed of poly(lactic-co-glycolic acid) (PLGA) knitted mesh combined with a collagen sponge impregnated with a gelatin hydrogel containing basic fibroblast growth factor (bFGF) [44]. The researchers implanted this construct into a rabbit tracheal defect and saw cartilage regeneration, epithelization, and preservation of tracheal patency while maintaining sufficient stiffness after six months [44]. These hybrid scaffolds provide improved mechanical stability and biocompatibility; however, long-term epithelial regeneration and integration remain inconsistent across experimental models. As previously mentioned, the challenge in developing a hybrid scaffold with synthetic and natural materials is incorporating materials that work together while minimizing host reaction.

More recently, decellularized extracellular matrix (ECM) scaffolds and recellularization strategies have emerged as promising approaches, as they can preserve native tracheal architecture and biomechanical organization while reducing immunogenicity [10,11,12]. Decellularization is the process of chemically or physically removing live cells from tissue, with the final product being an extracellular matrix scaffold [59]. Recent advances in decellularization and recellularization techniques have emphasized optimization of ECM protein preservation, vascular integration, stem cell repopulation, and scaffold remodeling as critical determinants of long-term graft success [60]. In vivo studies have demonstrated that ECM-containing scaffolds promote regeneration while reducing chronic inflammatory responses that cause secondary stenosis following tracheal repair [61,62,63]. Despite these advances, long-term epithelial maturation, coordinated tissue remodeling, vascular stability, and immune compatibility remain inconsistent across experimental models. In addition, decellularization of tissue remains labor-intensive and expensive, which makes this method less than ideal from an economic viewpoint.

Importantly, biomaterials are increasingly recognized as active regulators of cellular behavior and regenerative signaling and not simply as passive structural supports. Scaffold stiffness, porosity, degradation kinetics, and surface topography may directly influence stem cell differentiation, macrophage polarization, angiogenesis, epithelialization, and ECM remodeling [36,64]. Matrix elasticity has been shown to regulate stem cell lineage specification and cellular phenotype, highlighting the importance of the biomechanical properties of the material [65]. Likewise, biomaterial-induced immune responses significantly influence tissue remodeling through modulation of macrophage polarization and fibrotic signaling pathways [58]. Pro-inflammatory M1 macrophages promote cytokine release, fibrosis, ECM deposition, and graft failure, whereas pro-regenerative/anti-inflammatory M2 macrophages support angiogenesis, tissue remodeling, and scaffold integration [18,58,66]. Excessive M1 macrophage activation can lead to tracheal stenosis and impaired patency through chronic inflammatory remodeling. Consequently, researchers shifted focus to biomaterial strategies capable of modulating macrophage polarization toward pro-regenerative phenotypes (M2 phenotype) [18,66]. In addition, shape-memory biomaterials may further enable dynamic scaffold systems capable of adapting to physiologic airway movement and tissue remodeling over time [67]. These advances reflect a major conceptual shift in tracheal tissue engineering in which scaffold properties are leveraged as dynamic regenerative microenvironments capable of modulating stem cell behavior, immune responses, and long-term tissue integration.

**Figure 4:**
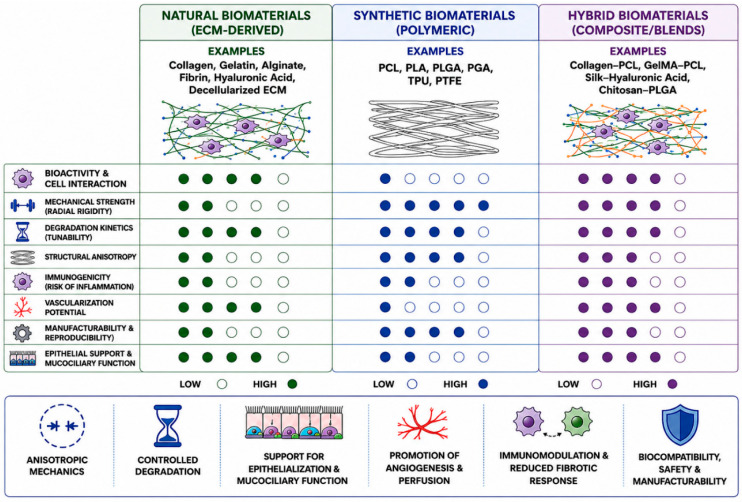
Comparative overview of natural, synthetic, and hybrid biomaterials used in tracheal tissue engineering and airway regeneration. Comparative scaffold characteristics, including bioactivity, rigidity, degradation, vascularization potential, immunogenicity, and manufacturability, are illustrated. The lower panel highlights key design requirements for next-generation tracheal scaffolds, including epithelial support, angiogenesis, immunomodulation, controlled biodegradation, and long-term biomechanical stability. Detailed ideas and instructions were entered into ChatGPT for assistance in creating this figure. The authors reviewed the figure and agreed with the information provided.

Future scaffold designs will likely require multifunctional biomaterials capable of simultaneously promoting epithelial regeneration, vascularization, immune modulation, and long-term biomechanical strength while remaining scalable, reproducible, and clinically translatable. Figure 4 depicts the three types of materials commonly used in tracheal tissue engineering while comparing various characteristics such as bioactivity, rigidity, and degradation.

## 5. Advanced Techniques for Tracheal Scaffold Development

Numerous scaffold fabrication techniques have been developed for facilitating tracheal repair and regeneration, including 3D bioprinting, 4D bioprinting, electrospinning, lyophilization, solvent casting/particulate leaching, gas foaming, fused deposition modeling (FDM), and selective laser sintering [68]. Each fabrication method possesses unique characteristics that may be advantageous depending on the biomaterial and intended scaffold architecture. These methods are categorized into conventional fabrication techniques and additive manufacturing techniques. Conventional techniques include solvent casting/particulate leaching, lyophilization, electrospinning, and gas foaming, whereas additive manufacturing approaches—also referred to as solid free-form fabrication—include 3D and 4D bioprinting, FDM, and selective laser sintering [68]. Conventional fabrication techniques are advantageous as they are well established, relatively cost-effective, and capable of producing highly porous scaffolds. However, these methods are often limited in their ability to precisely control internal architecture and create patient-specific constructs [68].

In contrast, additive manufacturing techniques enable fabrication of highly reproducible and customizable scaffolds based on Computer-Aided Design (CAD) models [68]. These approaches facilitate the development of patient-specific scaffolds using medical imaging data, thereby enhancing their translational potential for clinical applications. Nevertheless, additive manufacturing technologies remain under active development and require specialized engineering expertise and technical resources [68,69,70]. Supplemental Table S1 summarizes the major scaffold fabrication methods used in bioengineering and highlights advantages and limitations associated with each approach and how these methods can be used in tracheal tissue regeneration [71,72,73,74,75,76].

## 6. Leveraging Innate Repair Mechanisms in the Trachea

The trachea possesses innate repair mechanisms that can be leveraged for tissue engineering applications. One method includes the incorporation of the lateral tracheal vascular pedicle, which is a robust vascular network that provides nutrients, oxygen and immune support during periods of stress and injury [13]. Preservation of this network would be advantageous to regenerating scaffolds, as it would provide a needed vascular macro- and microenvironment enabling scaffold integration. Currently, researchers are looking into pre-vascularized scaffolds and angiogenic signaling as possible strategies.

In general, the tracheal epithelium exhibits a low turnover rate; however, following damage, quiescent resident progenitor cells rapidly activate and work to restore epithelial integrity [34,35]. When activated, basal stem cells undergo clonal expansion, proliferation and differentiation into different epithelial cells [26,35]. This regenerative potential makes basal cells particularly attractive for scaffold-based tissue engineering strategies. Currently, researchers are working on isolating epithelial cells from tracheal and bronchial biopsies to ultimately seed into a tissue-engineered trachea [30]. Butler et al. were able to culture human respiratory epithelial cells from endobronchial biopsies and demonstrated preserved mucociliary function [28]. This highlights the possibility of restoring mucociliary function by regenerating a functional tracheal epithelium within engineered scaffolds. In addition, this highlights the possibility of patient-specific epithelial engineering approaches for tracheal scaffolds.

Additionally, when the tracheal epithelium is compromised, the innate immune system works to repair the epithelium, leveraging signaling pathways such as Wnt/β-catenin, Notch, and transforming growth factor-beta (TGF-β) to promote repair, growth, and healing [24]. Wnt/β-catenin signaling contributes to epithelial regeneration and basal stem cell proliferation following injury, while Notch signaling plays a major role in epithelial lineage specification and differentiation [36]. During airway injury, activation of the Notch pathway promotes basal cell differentiation into goblet and secretory cell phenotypes required for restoration of epithelial homeostasis [31,36]. In contrast, dysregulated TGF-β signaling may contribute to fibrosis, excessive extracellular matrix deposition, and airway stenosis following scaffold implantation. Emerging evidence suggests that scaffold properties, including matrix stiffness, topography, and extracellular matrix composition, may directly influence these regenerative signaling pathways and leveraging these pathways may control cellular differentiation and tissue remodeling outcomes [36]. Consequently, biomaterials should not be viewed solely as passive structural supports but rather as active regulators of airway regeneration. Careful consideration of material stiffness and porosity design should be considered in tracheal engineering approaches.

## 7. The Future of Tracheal Regeneration

Future engineered tracheal scaffolds will benefit from a multilayered design leveraging the strength of both synthetic and natural biomaterials while incorporating key regenerative cells and ECM proteins. The use of 3D bioprinting technology and advanced bio-fabrication technologies has immense potential given its ability to personalize the design, create intricate scaffolds, and incorporate cells and ECM proteins within the construct. Using this technology, different scaffold layers can be bioprinted and implemented as a strategy to regenerate tracheal tissue. Through layer-by-layer assembly, different scaffold compartments can be optimized for distinct regenerative functions. For example, luminal regions may incorporate natural biomaterials seeded with epithelial progenitor and endothelial cells to promote epithelialization, mucociliary clearance, and angiogenesis, while outer structural layers may utilize mechanically reinforced synthetic polymers seeded with chondrocytes to support cartilage regeneration and tracheal stability. In addition, incorporating a bioreactor or implanting the scaffold into the host can promote vascular bed formation, thus providing the scaffold with a robust vascular supply prior to implantation in the trachea. Such multifunctional constructs would allow researchers to address several barriers to tracheal regeneration within a single engineered scaffold.

Lastly, future biomaterials will likely integrate immunomodulatory coatings, extracellular vesicles, controlled cytokine delivery systems, and macrophage-polarizing strategies to actively promote constructive remodeling while minimizing fibrosis and chronic inflammation [58]. Likewise, continued advances in airway stem cell biology and iPSC technologies may improve endothelial regeneration and patient-specific tissue engineering approaches while reducing immunologic incompatibility [19,35,51]. Targeted regulation of immune and healing responses will promote favorable scaffold incorporation and prevent the devastating consequences of rejection, such as tracheal stenosis, need for surgical repair, or tracheostomy creation.

## 8. Conclusions

Emerging evidence demonstrates that successful tracheal regeneration cannot rely solely on isolated scaffold systems or single-cell regenerative strategies. Future approaches will require multifunctional regenerative platforms that integrate advanced biomaterials, stem/progenitor cell populations, ECM signaling, immunomodulatory therapies, and patient-derived bio-fabrication technologies. Recent advances in 3D and 4D bioprinting, scaffold-free biofabrication, decellularized ECM engineering, immunology, and adaptive biomaterials have substantially expanded the potential for recreating physiologically functional tracheal tissue. Despite these advances, significant translational barriers persist. Furthermore, the highly publicized failures of prior tissue-engineered tracheal transplantation efforts underscore the importance of rigorous preclinical validation, reproducibility, and long-term safety assessments before widespread clinical implementation can be considered [77,78,79]. Addressing these challenges requires multidisciplinary approaches that integrate biomaterial engineering, stem cell biology, and regenerative biochemical signaling modulation, while remaining cognizant of past failures, in order to develop a clinically viable method of restoring functional tracheal tissue.

## Figures and Tables

**Table 1:** Major Biological and Engineering Barriers Associated with Tracheal Regeneration.

Challenge	Biological/Engineering Mechanism	Clinical Consequence	Current Strategies	Remaining Limitations
**Vascularization**	Limited angiogenesis and insufficient oxygen diffusion impair graft perfusion	Ischemia, necrosis, delayed healing, graft failure	VEGF delivery, endothelial cell incorporation, pre-vascularized scaffolds, in vivo bioreactors [12,18,19,20]	Poor long-term perfusion and unstable vascular integration
**Mechanical Stability**	Mechanical mismatch and insufficient radial rigidity impair airway support under physiologic loading	Airway collapse, stenosis, fatigue failure	Synthetic polymers, reinforced scaffolds, anisotropic and hybrid scaffold systems [17,18,21,22,23]	Balancing flexibility, durability, and tissue remodeling
**Immune Response**	Foreign body reaction, macrophage activation, fibrosis, and chronic inflammation	Fibrotic encapsulation, stenosis, graft rejection	Immunomodulatory biomaterials, cytokine delivery, extracellular vesicles, surface modification [17,24,25]	Persistent inflammation and incomplete immune regulation
**Epithelial Regeneration**	Inadequate epithelial differentiation and impaired mucociliary function	Infection, mucus accumulation, impaired airway clearance	Stem cell seeding, epithelial progenitor cells, organoids, bioactive coatings [12,26,27,28,29,30,31,32,33]	Incomplete epithelial maturation and long-term functionality
**Pediatric Growth Adaptation**	Static scaffolds fail to accommodate airway growth and remodeling	Growth restriction; repeated surgical intervention	Biodegradable and growth-compatible biomaterials, auxetic structures [10,11,16,24]	Limited long-term growth adaptability and durability

**Table 2:** Major Pseudostratified Epithelial Cell Populations and Their Functional Roles in Airway Regeneration.

Cell Type	Primary Function	Relevance to Tracheal Regeneration
**Ciliated Cells**	Coordinated ciliary beating mediates mucociliary clearance and removal of inhaled debris and pathogens [29]	Essential for restoring mucus transport and airway defense mechanisms
**Goblet Cells**	Production of mucus that traps inhaled particulates and pathogens [30]	Maintains airway lubrication and protective barrier function
**Basal Cells**	Multipotent stem/progenitor cells responsible for epithelial regeneration and differentiation [32]	Critical for long-term epithelial repair and scaffold repopulation
**Club (Clara) Cells**	Secretion of anti-inflammatory proteins and regulation of immune responses [33]	Supports epithelial repair and modulation of airway inflammation
**Pulmonary Neuroendocrine Cells (PNECs)**	Sensory and paracrine signaling regulating airway tone and responsiveness [27]	May influence epithelial repair and airway remodeling
**Tuft Cells**	Chemosensory signaling and regulation of mucociliary responses to irritants [37]	Contributes to epithelial defense and airway responsiveness
**Hillock Cells**	Protection of basal stem cell niches during injury and inflammation [35]	Supports preservation of regenerative epithelial capacity

**Table 3:** Cell types used in tracheal tissue engineering, along with their advantages and limitations.

Cell Type	Advantages	Limitations	Representative Study
**Mesenchymal Stem Cells (MSCs)**	Immunomodulatory, anti-inflammatory, multipotent, easy to isolate	Limited differentiation toward airway epithelium in some models	Shin et al. [50]
**Induced Pluripotent Stem Cells (iPSCs)**	High differentiation potential; can generate airway epithelial cells	Tumorigenicity concerns and complex differentiation protocols	Ikeda et al. [21]
**Autologous Chondrocytes**	Promote cartilage regeneration and structural support	Limited proliferation capacity and donor tissue requirements	Nomoto et al. [22]
**Airway Basal Stem Cells**	Native airway progenitors capable of regenerating epithelium	Difficult isolation and expansion	Lin et al. [35]
**Endothelial Cells**	Promote vascularization of scaffolds	Require supportive microenvironment for stability	Khalid et al. [41]

**Table 4:** Summary of Biomaterials Used in Tracheal Tissue Engineering and Their Advantages and Limitations.

Material	Type	Strengths	Limitations	Representative Study
**Alginate**	Natural	Biocompatible; supports cell adhesion and hydrogel formation	Weak mechanical strength and rapid degradation	Luo et al. [53]
**Chitosan**	Natural	Antimicrobial properties; promotes cell attachment	Limited mechanical stability	Nematollahi et al. [54]
**Collagen**	Natural	Mimics extracellular matrix; excellent cell compatibility	Rapid degradation and poor structural strength	Xu et al. [55]
**Fibrin**	Natural	Supports cell infiltration and angiogenesis	Weak mechanical properties and fast degradation	Dai et al. [56]
**Gelatin**	Natural	Promotes cell adhesion and proliferation	Low mechanical stability	Fares et al. [20]
**Hyaluronic Acid**	Natural	Supports cartilage regeneration and ECM signaling	Limited structural strength	Xu et al. [55]
**Soy Protein**	Natural	Biodegradable and supportive for cell growth	Limited studies in airway applications	Naik et al. [6]
**HDPE**	Synthetic	High mechanical strength and durability	Poor biodegradability and limited bioactivity	Naik et al. [6]
**PLA**	Synthetic	Biodegradable; tunable degradation rate	Can produce acidic degradation byproducts	DeStefano et al. [52]
**PCL**	Synthetic	Excellent mechanical strength; slow degradation	Hydrophobic surface limits cell attachment	Gandha et al. [42]
**PC**	Synthetic	Strong structural support	Limited biodegradability	Artham et al. [43]
**PET**	Synthetic	High durability and stability	Limited biocompatibility in regenerative applications	Naik et al. [6]
**PGA**	Synthetic	Biodegradable with good mechanical strength	Rapid degradation may compromise structural support	Naik et al. [6]
**PLGA**	Synthetic	Tunable degradation and widely used in tissue engineering	Degradation may produce acidic environment	Tatekawa et al. [44]
**PPE**	Synthetic	Tunable degradation and polymer properties	Limited long-term airway studies	Naik et al. [6]
**TPU**	Synthetic	Elastic and mechanically durable	Poor bioactivity for cell attachment	Naik et al. [6]

Abbreviations: HDPE—high-density polyethylene; PLA—polylactic acid; PCL—polycaprolactone; PC—polycarbonate; PET—polyethylene terephthalate; PGA—polyglycolic acid; PLGA—poly(lactic-co-glycolic) acid; PPE—polyphosphoesters; TPU—thermoplastic polyurethane.

## Data Availability

Given that this paper is a review article, this is not applicable. This manuscript does not report data generation or analysis.

## Supplementary Materials

The following supporting information can be downloaded at: https://www.mdpi.com/article/10.3390/bioengineering13060641/s1, Supplementary Table S1: Description and Comparison of Conventional Versus Additive Manufacturing Techniques with Considerations for Tracheal Tissue Engineering.

## Abbreviations

The following abbreviations are used in this manuscript:

TEF	Tracheoesophageal fistula
VEGFs	Vascular endothelial growth factors
TGF-β	Transforming growth factor-beta
MSC	Mesenchymal stem cell
iPSCs	Induced pluripotent stem cells
PLA	Polylactic acid
PCL	Polycaprolactone
PC	Polycarbonate
TPU	Thermoplastic polyurethane
PGA	Polyglycolic acid
PLGA	Poly(lactic-co-glycolic) acid
PPE	Polyphosphoesters
PET	Polyethylene terephthalate
HDPE	High-density polyethylene
bFGF	Basic fibroblast growth factor
ECM	Extracellular matrix
3D	Three-dimension
4D	Four-dimension
FDM	Fused deposition modeling
PNECs	Pulmonary neuroendocrine cells

## References

[1] Herrera M.A., Tintinago L.F., Victoria Morales W., Ordonez C.A., Parra M.W., Betancourt-Cajiao M., Caicedo Y., Guzman-Rodriguez M., Gallego L.M., Gonzalex Hadad A. (2020). Damage control of laryngotracheal trauma: The golden day. Colomb. Med..

[2] Grewal H.S., Dangayach N.S., Ahmad U., Ghosh S., Gildea T., Mehta A.C. (2019). Treatment of Tracheobronchial Injuries: A Contemporary Review. Chest.

[3] De Jong R., Hohman M.H., Farahani C. (2025). Laryngeal Clefts. StatPearls [Internet].

[4] Asnaghi A., Macchiarini P., Mantero S. (2009). Tissue engineering toward organ replacement: A promising approach in airway transplant. Int. J. Artif. Organs.

[5] Auchincloss H.G., Wright C.D. (2016). Complications after tracheal resection and reconstruction: Prevention and treatment. J. Thorac. Dis..

[6] Naik S.S., Dutta N.K., Sukumaran Nair K., Choudhury N.R. (2025). Critical advances in biofabrication and biomaterial strategies in tracheal tissue engineering: A comprehensive overview. Adv. Colloid Interface Sci..

[7] Tapias L.F., Mathisen D.J. (2018). Prevention and management of complications following tracheal resections-lessons learned at the Massachusetts General Hospital. Ann. Cardiothorac. Surg..

[8] Wei S., Zhang Y., Luo F., Duan K., Li M., Lv G. (2024). Tissue-engineered tracheal implants: Advancements, challenges, and clinical considerations. Bioeng. Transl. Med..

[9] Sadreameli S.C., McGrath-Morrow S.A. (2016). Respiratory Care of Infants and Children with Congenital Tracheo-Oesophageal Fistula and Oesophageal Atresia. Paediatr. Respir. Rev..

[10] Edwards N.A., Shacham-Silverberg V., Weitz L., Kingma P.S., Shen Y., Wells J.M., Chung W.K., Zorn A.M. (2021). Developmental basis of trachea-esophageal birth defects. Dev. Biol..

[11] Faure S., de Santa Barbara P. (2011). Molecular embryology of the foregut. J. Pediatr. Gastroenterol. Nutr..

[12] Jacobsen B., VanKampen N., Ashurst J.V. (2025). Anatomy, Head and Neck, Thyrohyoid Membrane. StatPearls.

[13] Yeou S.H., Shin Y.S. (2025). Tissue-Engineered Tracheal Reconstruction. Biomimetics.

[14] Mammana M., Bonis A., Verzeletti V., Dell’Amore A., Rea F. (2024). Tracheal Tissue Engineering: Principles and State of the Art. Bioengineering.

[15] Mieczkowski B., Seavey B.F. (2023). Anatomy, Head and Neck, Trachea. StatPearls [Internet].

[16] Kishimoto K., Morimoto M. (2021). Mammalian tracheal development and reconstruction: Insights from in vivo and in vitro studies. Development.

[17] Shamji F.M. (2018). Factors Favoring and Impairing Healing of Tracheal Anastomosis. Thorac. Surg. Clin..

[18] Anderson J.M., Rodriguez A., Chang D.T. (2008). Foreign body reaction to biomaterials. Semin. Immunol..

[19] Imaizumi M., Nomoto Y., Sato Y., Sugino T., Miyake M., Wada I., Nakamura T., Omori K. (2013). Evaluation of the use of induced pluripotent stem cells (iPSCs) for the regeneration of tracheal cartilage. Cell Transpl..

[20] Fares M.M., Shirzaei Sani E., Portillo Lara R., Oliveira R.B., Khademhosseini A., Annabi N. (2018). Interpenetrating network gelatin methacryloyl (GelMA) and pectin-g-PCL hydrogels with tunable properties for tissue engineering. Biomater. Sci..

[21] Ikeda M., Imaizumi M., Yoshie S., Otsuki K., Miyake M., Hazama A., Wada I., Omori K. (2016). Regeneration of tracheal epithelium using mouse induced pluripotent stem cells. Acta Otolaryngol..

[22] Nomoto M., Nomoto Y., Tada Y., Tani A., Otsuki K., Suzuki R., Nakamura T., Omori K. (2013). Bioengineered trachea using autologous chondrocytes for regeneration of tracheal cartilage in a rabbit model. Laryngoscope.

[23] Gomez-Florit M., Pardo A., Domingues R.M.A., Graça A.L., Babo P.S., Reis R.L., Gomes M.E. (2020). Natural-Based Hydrogels for Tissue Engineering Applications. Molecules.

[24] Safshekan F., Tafazzoli-Shadpour M., Abdouss M., Behgam Shadmehr M., Ghorbani F. (2017). Investigation of the Mechanical Properties of the Human Tracheal Cartilage. Tanaffos.

[25] Cao Y., Cheng P., Sang S., Xiang C., An Y., Wei X., Shen Z., Zhang Y., Li P. (2021). Mesenchymal stem cells loaded on 3D-printed gradient poly(ε-caprolactone)/methacrylated alginate composite scaffolds for cartilage tissue engineering. Regen. Biomater..

[26] Rock J.R., Randell S.H., Hogan B.L. (2010). Airway basal stem cells: A perspective on their roles in epithelial homeostasis and remodeling. Dis. Models Mech..

[27] Thakur A., Mei S., Zhang N., Zhang K., Taslakjian B., Lian J., Wu S., Chen B., Solway J., Chen H.J. (2024). Pulmonary neuroendocrine cells: Crucial players in respiratory function and airway-nerve communication. Front. Neurosci..

[28] Butler C.R., Hynds R.E., Gowers K.H., Lee Ddo H., Brown J.M., Crowley C., Teixeira V.H., Smith C.M., Urbani L., Hamilton N.J. (2016). Rapid Expansion of Human Epithelial Stem Cells Suitable for Airway Tissue Engineering. Am. J. Respir. Crit. Care Med..

[29] Bustamante-Marin X.M., Ostrowski L.E. (2017). Cilia and Mucociliary Clearance. Cold Spring Harb. Perspect. Biol..

[30] Rogers D.F. (2003). The airway goblet cell. Int. J. Biochem. Cell Biol..

[31] Rogers D.F. (1994). Airway goblet cells: Responsive and adaptable front-line defenders. Eur. Respir. J..

[32] Ruysseveldt E., Martens K., Steelant B. (2021). Airway Basal Cells, Protectors of Epithelial Walls in Health and Respiratory Diseases. Front. Allergy.

[33] Davis J.D., Wypych T.P. (2021). Cellular and functional heterogeneity of the airway epithelium. Mucosal Immunol..

[34] Eenjes E., Tibboel D., Wijnen R.M.H., Rottier R.J. (2022). Lung epithelium development and airway regeneration. Front. Cell Dev. Biol..

[35] Lin B., Shah V.S., Chernoff C., Sun J., Shipkovenska G.G., Vinarsky V., Waghray A., Xu J., Leduc A.D., Hintschich C.A. (2024). Airway hillocks are injury-resistant reservoirs of unique plastic stem cells. Nature.

[36] Chen Y., Tang H., Zhang Y., Wang L., Zhu J., Wang L., Li A., Zeng X., Yin B., Liang Y. (2025). Multiplexed self-adaptable Janus hydrogels rescue epithelial malfunction to promote complete trachea repair. Nat. Commun..

[37] Hollenhorst M.I., Husnik T., Zylka M., Duda N., Flockerzi V., Tschernig T., Maxeiner S., Krasteva-Christ G. (2023). Human airway tuft cells influence the mucociliary clearance through cholinergic signalling. Respir. Res..

[38] Liu L., Dharmadhikari S., Pouliot R.A., Li M.M., Minneci P.M., Tan Z., Shontz K., Johnson J., Reynolds S.D., Breuer C.K. (2021). Modulation of Synthetic Tracheal Grafts with Extracellular Matrix Coatings. Bioengineering.

[39] Howat W.J., Holmes J.A., Holgate S.T., Lackie P.M. (2001). Basement membrane pores in human bronchial epithelium: A conduit for infiltrating cells?. Am. J. Pathol..

[40] Downey R.P., Samra N.S. (2026). Anatomy, Thorax, Tracheobronchial Tree. StatPearls.

[41] Khalid T., Soriano L., Lemoine M., Cryan S.A., O’Brien F.J., O’Leary C. (2023). Development of tissue-engineered tracheal scaffold with refined mechanical properties and vascularisation for tracheal regeneration. Front. Bioeng. Biotechnol..

[42] Gandha P., Surve T., Kandasubramanian B., Mozafari M., Singh Chauhan N.P. (2023). 15—Polycaprolactone as biomaterial. Handbook of Polymers in Medicine.

[43] Artham T., Doble M. (2008). Biodegradation of aliphatic and aromatic polycarbonates. Macromol. Biosci..

[44] Tatekawa Y., Kawazoe N., Chen G., Shirasaki Y., Komuro H., Kaneko M. (2010). Tracheal defect repair using a PLGA-collagen hybrid scaffold reinforced by a copolymer stent with bFGF-impregnated gelatin hydrogel. Pediatr. Surg. Int..

[45] Di Cicco M., Kantar A., Masini B., Nuzzi G., Ragazzo V., Peroni D. (2021). Structural and functional development in airways throughout childhood: Children are not small adults. Pediatr. Pulmonol..

[46] Szpinda M., Daroszewski M., Woźniak A., Szpinda A., Mila-Kierzenkowska C. (2012). Tracheal dimensions in human fetuses: An anatomical, digital and statistical study. Surg. Radiol. Anat..

[47] Luscan R., Leboulanger N., Fayoux P., Kerner G., Belhous K., Couloigner V., Garabedian E.N., Simon F., Denoyelle F., Thierry B. (2020). Developmental changes of upper airway dimensions in children. Paediatr. Anaesth..

[48] Margiana R., Markov A., Zekiy A.O., Hamza M.U., Al-Dabbagh K.A., Al-Zubaidi S.H., Hameed N.M., Ahmad I., Sivaraman R., Kzar H.H. (2022). Clinical application of mesenchymal stem cell in regenerative medicine: A narrative review. Stem Cell Res. Ther..

[49] Hernández R., Jiménez-Luna C., Perales-Adán J., Perazzoli G., Melguizo C., Prados J. (2020). Differentiation of Human Mesenchymal Stem Cells towards Neuronal Lineage: Clinical Trials in Nervous System Disorders. Biomol. Ther..

[50] Shin Y.S., Choi J.W., Park J.K., Kim Y.S., Yang S.S., Min B.H., Kim C.H. (2015). Tissue-engineered tracheal reconstruction using mesenchymal stem cells seeded on a porcine cartilage powder scaffold. Ann. Biomed. Eng..

[51] Aboul-Soud M.A.M., Alzahrani A.J., Mahmoud A. (2021). Induced Pluripotent Stem Cells (iPSCs)-Roles in Regenerative Therapies, Disease Modelling and Drug Screening. Cells.

[52] DeStefano V., Khan S., Tabada A. (2020). Applications of PLA in modern medicine. Eng. Regen..

[53] Luo Y., Lode A., Gelinsky M. (2013). Direct Plotting of Three-Dimensional Hollow Fiber Scaffolds Based on Concentrated Alginate Pastes for Tissue Engineering. Adv. Healthc. Mater..

[54] Nematollahi Z., Tafazzoli-Shadpour M., Zamanian A., Seyedsalehi A., Mohammad-Behgam S., Ghorbani F., Mirahmadi F. (2017). Fabrication of Chitosan Silk-based Tracheal Scaffold Using Freeze-Casting Method. Iran. Biomed. J..

[55] Xu Y., Wang Z., Hua Y., Zhu X., Wang Y., Duan L., Zhu L., Jiang G., Xia H., She Y. (2021). Photocrosslinked natural hydrogel composed of hyaluronic acid and gelatin enhances cartilage regeneration of decellularized trachea matrix. Mater. Sci. Eng. C Mater. Biol. Appl..

[56] Dai Y., Liu G., Ma L., Wang D., Gao C. (2016). Cell-free macro-porous fibrin scaffolds for in situ inductive regeneration of full-thickness cartilage defects. J. Mater. Chem. B.

[57] Novosel E.C., Kleinhans C., Kluger P.J. (2011). Vascularization is the key challenge in tissue engineering. Adv. Drug Deliv. Rev..

[58] Brown B.N., Sicari B.M., Badylak S.F. (2014). Rethinking regenerative medicine: A macrophage-centered approach. Front. Immunol..

[59] Crapo P.M., Gilbert T.W., Badylak S.F. (2011). An overview of tissue and whole organ decellularization processes. Biomaterials.

[60] Gomes K.T., Ranga Prasad P., Singh Sandhu J., Kumar A., Kumar N.A.N., Shridhar N.B., Bisht B., Paul M.k. (2025). Decellularization techniques: Unveiling the blueprint for tracheal tissue engineering. Front. Bioeng. Biotechnol..

[61] Pouliot R.A., Link P.A., Mikhai N.S., Schneck M.B., Valentine M.S., Kamga Gninzeko F.J., Herbert J.A., Sakagami M., Heise R.L. (2016). Development and characterization of a naturally derived lung extracellular matrix hydrogel. J. Biomed. Mater. Res. A.

[62] Villegas-Alvarez F., González-Zamora J.F., González-Maciel A., Soriano-Rosales R., Pérez-Guille B., Padilla-Sánchez L., Reynoso-Robles R., Ramos-Morales A., Zenteno-Galindo E., Pérez-Torres A. (2010). Fibrocollagen-covered prosthesis for a noncircumferential segmental tracheal replacement. J. Thorac. Cardiovasc. Surg..

[63] Young B.M., Shankar K., Allen B.P., Pouliot R.A., Schneck M.B., Mikhaiel N.S., Heise R.L. (2017). Electrospun Decellularized Lung Matrix Scaffold for Airway Smooth Muscle Culture. ACS Biomater. Sci. Eng..

[64] Reyes C.D., Petrie T.A., García A.J. (2008). Mixed extracellular matrix ligands synergistically modulate integrin adhesion and signaling. J. Cell Physiol..

[65] Engler A.J., Sen S., Sweeney H.L., Discher D.E. (2006). Matrix elasticity directs stem cell lineage specification. Cell.

[66] Liang Y., Wei S., Zhang A. (2025). Bioengineered tracheal graft with enhanced vascularization and mechanical stability for functional airway reconstruction. Regen. Ther..

[67] Zhang X., Yang Y., Yang Z., Ma R., Aimaijiang M., Xu J., Zhang Y., Zhou Y. (2023). Four-Dimensional Printing and Shape Memory Materials in Bone Tissue Engineering. Int. J. Mol. Sci..

[68] Suamte L., Tirkey A., Barman J., Jayasekhar B. (2023). Various manufacturing methods and ideal properties of scaffolds for tissue engineering applications. Smart Mater. Manuf..

[69] Papaioannou T.G., Manolesou D., Dimakakos E., Tsoucalas G., Vavuranakis M., Tousoulis D. (2019). 3D Bioprinting Methods and Techniques: Applications on Artificial Blood Vessel Fabrication. Acta Cardiol. Sin..

[70] Ashammakhi N., Ahadian S., Zengjie F., Suthiwanich K., Lorestani F., Orive G., Ostrovidov S., Khademhosseini A. (2018). Advances and Future Perspectives in 4D Bioprinting. Biotechnol. J..

[71] Hutmacher D.W. (2000). Scaffolds in tissue engineering bone and cartilage. Biomaterials.

[72] Katrilaka C., Karipidou N., Petrou N., Manglaris C., Katrilakas G., Tzavellas A.N., Pitou M., Tsiridis E.E., Choli-Papadopoulou T., Aggeli A. (2023). Freeze-Drying Process for the Fabrication of Collagen-Based Sponges as Medical Devices in Biomedical Engineering. Materials.

[73] Zulkifli M.Z.A., Nordin D., Shaari N., Kamarudin S.K. (2023). Overview of Electrospinning for Tissue Engineering Applications. Polymers.

[74] Dehghani F., Annabi N. (2011). Engineering porous scaffolds using gas-based techniques. Curr. Opin. Biotechnol..

[75] Roskies M., Jordan J.O., Fang D., Abdallah M.N., Hier M.P., Mlynarek A., Tamimi F., Tran S.D. (2016). Improving PEEK bioactivity for craniofacial reconstruction using a 3D printed scaffold embedded with mesenchymal stem cells. J. Biomater. Appl..

[76] Neishabouri A., Soltani Khaboushan A., Daghigh F., Kajbafzadeh A.M., Majidi Zolbin M. (2022). Decellularization in Tissue Engineering and Regenerative Medicine: Evaluation, Modification, and Application Methods. Front. Bioeng. Biotechnol..

[77] Delaere P.R., Van Raemdonck D. (2014). The trachea: The first tissue-engineered organ?. J. Thorac. Cardiovasc. Surg..

[78] Elliott M.J., De Coppi P., Speggiorin S., Roebuck D., Butler C.R., Samuel E., Crowley C., McLaren C., Fierens A., Vondrys D. (2012). Stem-cell-based, tissue engineered tracheal replacement in a child: A 2-year follow-up study. Lancet.

[79] Grillo H.C. (2003). Development of tracheal surgery: A historical review. Part 2: Treatment of tracheal diseases. Ann. Thorac. Surg..

