# Supplementary material for: Tracheal Tissue Engineering: Advances and Challenges
Source: Bioengineering (Basel). 2026 May 29;13(6):641. doi: 10.3390/bioengineering13060641 (PMC13296255; doi:10.3390/bioengineering13060641)
Supplement: Supplementary file 1 [file bioengineering-13-00641-s001.zip › bioengineering-4303790-supplementary.pdf]

## Supplemental Material

**Supplementary Table S1.** Description and Comparison of Conventional Versus Additive Manufacturing Techniques with Considerations for Tracheal Tissue Engineering

| | Technique Name | Description | Common Materials | How Cells are Incorporated | Porosity | Advantage | Disadvantage | Tracheal Tissue Engineering Considerations | References |
|---|---|---|---|---|---|---|---|---|---|
| Conventional | Solvent casting/particulate leaching | Dissolving a polymer into a solvent with insoluble salt following evaporation of the solvent and leaching out the salt | Polymers | Seeding | Highly | Cheap, Customizable pore size | Length, Toxic Solvents | Creation of a porous scaffold for cell growth, typically used in cartilage applications thus advantageous for cartilage rings | Suamte et al. [68], Hutmacher et al. [71] |
| | Lyophilization | Freezing a polymer following sublimation in a vacuum system | Temperature controlled polymers | Seeding, Embedment (depending on solvents) | Highly | Good for materials sensitive to high temperatures, Customizable pore size, Interconnected pores | Cytotoxic Solvents | Creation of a porous scaffold for cell growth | Suamte et al.[68], Katrilaka et al. [72] |
| | Electrospinning | Electrostatic force in combination with rotation to create nanofibers | Polymers | Seeding, Embedment | Highly | Nanofiber structures, high tensile strength | Toxic solvents, Poor control over size and shape of pores | Allows for high tensile strength that could withstand various forces during respiration | Suamte et al. [68], Zulkifli et al. [73] |
| | Gas Foaming | Foaming agent added to polymer to generate an insert gas to create pores | Polymers | Seeding, Embedment | High | Cheap, Inert gas utilized, | Very high temperatures, Surface layer not porous, pores not interconnected | Advantageous to develop externally smooth cartilage rings while retaining internal porosity for cell growth and proliferation | Suamte et al. [68], Dehghani et al. [74] |
| Additive Manuf | 3D Bioprinting | Polymer ink deposited through extrusion | Polymers | Seeding, Embedment | Variable depending on polymer | Detailed Structures, Reproducibility | Post-processing, Expensive, Printer head clogging | Utilize CAD files for patient specific anatomy | Suamte et al. [68], Papaioannou et al. [69] |

| | | | | | | | | | |
|---|---|---|---|---|---|---|---|---|---|
| | | nozzle via pressure layer by layer | | | | | | | |
| | 4D Bioprinting | Similar to 3D printing with application of an additional external stimulus to allow the structure to change shape | Polymers | Seeding, Embedment | Variable depending on polymer | Detailed Structures | Post-processing, Expensive, Printer head clogging | Utilize CAD files for patient specific anatomy, Potential to be used in pediatric trachea scaffolds | Suamte et al. [68], Ashammakhi et al. [70] |
| | Fused Deposition Modeling | Polymer melted and deposited through a hot extrusion nozzle | Thermoplastic Polymers, Ceramic | Seeding | Highly | Good mechanical strength of final scaffold, No cytotoxic agents | Limited applicability for biodegradable polymers | Utilize CAD files for patient specific anatomy | Suamte et al. [68] |
| | Selective Laser Sintering | Lasers sinter powder particles into thin layers | Metal, Ceramic, Polymers | Seeding | Moderate to High | Internal porosity with interconnectivity | Slow process, Post-processing, Expensive, High temperatures | Advantageous to develop externally smooth cartilage rings while retaining internal porosity for cell growth and proliferation | Suamte et al. [68], Roskies et al. [75] |
| | Decellularization | Breakdown of cell membranes through chemical, physical or enzymatic methods with cellular components removed | Native Tissue | Seeding | Depends on ECM of decellularized tissue | Preserved natural microenvironments | Numerous protocols specific to various tissues, Difficulty reintroducing cells, Contamination | Incorporated into a multicompartment scaffold to assist in cell adhesion and regeneration | Suamte et al [68], Neishabouri et al. [76] |

**Abbreviations:** CAD – Computer-Aided Design; 3D – Three-dimension; 4D – Four-dimension
